# Parental insulin resistance is associated with unhealthy lifestyle behaviours independently of body mass index in children: The Feel4Diabetes study

**DOI:** 10.1007/s00431-022-04449-0

**Published:** 2022-03-26

**Authors:** Esther M. González-Gil, Natalia Giménez-Legarre, Greet Cardon, Christina Mavrogianni, Jemina Kivelä, Violeta Iotova, Tsvetalina Tankova, Rurik Imre, Stavros Liatis, Konstantinos Makrilakis, Peter Schwarz, Patrick Timpel, Elisabeth Dupont, Pedro Couck, Yannis Manios, Luis A. Moreno

**Affiliations:** 1 Department of Biochemistry and Molecular Biology II, Center of Biomedical Research (CIBM), Instituto de Nutrición y Tecnología de los Alimentos, Universidad de Granada, Granada, Spain; 2 Growth, Exercise, Nutrition and Development (GENUD) Research Group, University of Zaragoza, Edif. del SAI (Servicio de Apoyo a la Investigación), C/ Pedro Cerbuna 12, 50009 Zaragoza, Spain; 3 CIBER Fisiopatología de la Obesidad y Nutrición, Instituto de Salud Carlos III, Madrid, Spain; 4 Instituto Agroalimentario de Aragón (IA2), Instituto de Investigación Sanitaria Aragón (IIS Aragón), Zaragoza, Spain; 5 Department of Movement and Sports Sciences, Ghent University, Ghent, Belgium; 6 Department of Nutrition and Dietetics, School of Health Sciences & Education, Harokopio University, Athens, Greece; 7 Public Health Promotion Unit, Finnish Institute for Health and Welfare, Helsinki, Finland; 8 Department of Pediatrics, Medical University of Varna, Varna, Bulgaria; 9 Department of Endocrinology, Medical University of Sofia, Sofia, Bulgaria; 10 Department of Family and Occupational Medicine, University of Debrecen, Debrecen, Hungary; 11 Medical School, National and Kapodistrian University, Athens, Greece; 12 Department of Prevention and Care of Diabetes, Technical University Dresden, Dresden, Germany; 13 International Diabetes Federation, Brussels, Belgium; 14 Cerba Healthcare Belgium, Ghent, Belgium

**Keywords:** Insulin resistance, Lifestyle, Family behaviours, Children, European

## Abstract

Parental health is associated with children’s health and lifestyles. Thus, the aim of the present study was to assess lifestyle behaviours of children of parents with insulin resistance (IR) and at risk of type 2 diabetes. 2117 European families from the Feel4Diabetes-study were identified as being at risk for diabetes with the FINDRISC questionnaire and included in the present study. One parent and one child per family were included. Parental IR was considered when homeostasis model assessment (HOMA) was equal or higher than 2.5. Children’s screen-time, physical activity and diet were assessed and clustered by *K*-means. Weight and height were measured and children’s body mass index (BMI) was calculated. For children, a Healthy Diet Score (HDS) was calculated. Linear regression and multilevel logistic regression analyses were performed to assess the associations between parental IR and children’s lifestyle behaviours in 2021. Children of parents with IR had higher BMI (*p* < 0.001) and spent more screen time (*p* = 0.014) than those of non-IR parents. Children of parents with IR had a lower value in the breakfast and vegetable components of the HDS (*p* = 0.008 and *p* = 0.05). Four lifestyle clusters were found. Children of IR parents had higher odds of being in a non-healthy cluster (OR: 1.19; 95%CI: 1.001–1.437).

*Conclusion*: Having an IR parent was associated with a high screen time and an increased probability of having an unhealthy lifestyle pattern in children. These data point out that children’s lifestyles should be assessed in families with IR parents to provide tailored interventions.

**Table:** 

**What is Known:** *• Children with diabetic or insulin-resistant parents could also develop this condition.* *• Unhealthy lifestyles are directly related with insulin resistance even in children.*	
**What is New:** *• Children from parents with insulin resistance have higher chances of unhealthy lifestyles.* *• A higher BMI was found for those children with an insulin-resistant parent.*

**Supplementary information:**

The online version contains supplementary material available at 10.1007/s00431-022-04449-0.

## Introduction

Type 2 diabetes (T2D) is globally an important cause of morbidity and mortality [1]. In 2014, it was reported that 422 million adults had diabetes [2]. However, this estimation is projected to rise to 642 million by 2040 [1]. Nowadays, according to the International Diabetes Federation, 9.3% of the global adult population is estimated to be living with diabetes [3].

Type 2 diabetes is associated with lifestyle behaviours such as sedentary behaviours, physical inactivity, consumption of high-energy foods and other as yet unknown factors [3]. Among all risk factors for T2D, family history of diabetes consistently confirms an increased risk for T2D in both cross-sectional and longitudinal studies [4, 5].

A state of pre-diabetes is insulin resistance (IR), which refers to whole-body reduced glucose uptake in response to physiological insulin levels [6]. It has been observed that children of parents with IR have significantly higher values of insulin resistance and obesity than those children of parents without IR [7]. This could be explained, at least in part, by genetics as there are more than 120 genetic loci discovered that have been associated with T2D or glucose and insulin concentrations in European and multi-ethnic populations [8]. However, in adults, there is no consensus on the cut-offs that aim to assess IR [9]. The most widely used was proposed by Matthews et al. [10], considering IR when the HOMA-IR index is higher than 2.5.

Associations between IR and lifestyle behaviours have been observed even in children [11]. The main risk factors for pre-diabetes in children and adolescents are parental diabetes, pubertal stage and obesity [12]. Also, parents have a strong influence on the establishment of their children’s lifestyle-related behaviours [13]. Some studies showed a positive relationship between different behaviours of parents and those of their children, as physical activity [14], screen time [14], food preferences and eating behaviours [15]. It has been observed that there are patterns of lifestyle behaviours related with energy balance that may co-occur in sub-groups of children [16–18]. Among these patterns, the unhealthy one is usually characterized by unhealthy dietary habits (e.g. high intake of unhealthy snacks and/or sugary products among others), high levels of sedentary behaviour (specially related to screen use) and low levels of physical activity.

Different studies also suggest that unhealthy lifestyle behaviours could be related to a higher risk of IR in children and the development of type 2 diabetes [19, 20]. However, to our knowledge, this is the first study to assess children’s lifestyle behaviours depending on parental IR status.

Thus, the main objective of the present study is to assess lifestyle behaviours, specifically, diet, screen time and physical activity, of children depending on their parents’ IR status in European families at high risk of developing type 2 diabetes.

## Methods

### Study design and data collection

The Feel4Diabetes-study was an intervention study aiming to develop, implement and evaluate an evidence-based and potentially cost-effective and scalable intervention programme to prevent type 2 diabetes across Europe, primarily focusing on families from vulnerable groups, i.e. those from low socioeconomic status or at exclusion risk. The Feel4Diabetes-study was conducted between 2015 and 2019 in six European countries representing high-income countries (Belgium and Finland), low-income countries (Bulgaria and Hungary) and countries under austerity measures (Greece and Spain). A detailed description of the Feel4Diabetes-study has been previously published [21].

In the Feel4Diabetes-study, children attending the first three grades of compulsory education as well as their parents were recruited to the study. 11,396 families were included in the study and parents were screened for type 2 diabetes risk using the FINDRISC questionnaire, a tool developed to identify subjects at high risk of T2D [21]. FINDRISC score was obtained based on eight questions related to age, waist circumference (WC), weight, height, consumption of fruit and vegetables, physical activity, history of high blood glucose, family history of diabetes and the use of antihypertensive medication. A family was regarded as “high-risk” if at least one parent fulfilled the country-specific cut-off point for FINDRISC. Parents identified as at high risk of type 2 diabetes were invited to participate in the second-stage screening which included a brief medical check-up. 2537 high-risk families were identified and measured at baseline.

Out of those identified as high-risk families, 2117 families were included in the present study. Inclusion criteria were having complete biochemical data: total cholesterol (TC), triglycerides (TG), LDL cholesterol (LDL-c) and HDL cholesterol (HDL-c), Glucose and insulin and blood pressure data from the parents while having information regarding screen time, physical activity, diet and anthropometric indices (weight and height) from the child. Family dyads were included in this study, i.e. one parent and one child from each family. In the case of families with more than one child, children were randomly selected. In families with both parents at risk according to FINDRISC, parents were randomly selected for each family. However, for those families with only one parent at risk, that parent was included.

The Feel4Diabetes-study adhered to the Declaration of Helsinki and was approved by each local ethical committee. Participants received an information letter in which they were informed about the purpose of the study. Written and signed informed consent was obtained from the parents/caregivers.

### Anthropometric indices

Weight was measured twice in light indoor clothes and without shoes with a calibrated scale (Type SECA 813). Body height was measured twice with a wall-mounted stadiometer (Type SECA 217). If the difference between the two measurements was greater than 0.1 cm or 0.1 kg, a third assessment was carried out. Body mass index (BMI) was calculated from height and weight (kg/m2).

### Physical activity and screen-time

The family’s energy balance-related behaviours’ questionnaire was fulfilled by one of the parents or caregivers. Parents’ and children’s physical activity was assessed using the following question: “In the previous week, how many days were you/was your child active for at least 30 min/day (parent)/ 60 min/day (child) (a) on weekdays, and (b) on weekend days?”. The term “active” means any type of movement or physical activity that increases your heart rate or makes you sweat a little for example cycling, dancing, gardening, fitness, etc. For weekdays, there was a 6-point scale ranging from “none” to “five days”. For weekend days, there were three possible answers ranging from “none” to “two days”. The information was categorized into days per week.

Regarding screen time, i.e. all activities related to TV/DVD-watching, use of PC, smartphone, tablet or playing video games, parents’ and children’s screen-time was assessed using the following question for both, weekdays and weekend days: “About how many hours per day do you usually spend on screen activities (excluding work/school) on weekdays, and (b) on weekend days?”. There was a 10-point scale ranging from “None” to “more than 7 h”, with 30-min and 1-h intervals among the responses.

### Parental education

It was obtained by a self-administered questionnaire completed by parents. This was asked in a scale question ranging from “less than 6 years” to “more than 16 years” of education.

### Feel4Diabetes healthy diet score

A Healthy Diet Score (HDS) for adults was developed by Virtanen et al. [22]. HDS components were developed according to the 14 diet-related questions in the Feel4Diabetes questionnaire and 12 components were measured: breakfast, vegetables, fruits and berries, sugary drinks, whole-grain cereals, low-fat dairy, nuts and seeds, oils and fats, red meat, sweet snacks, salty snacks and family meals. Each component included one or two questions about intake frequencies of each food group or dietary behaviour, i.e. having breakfast or family meals. For this study, we adapted the score for adults with the information available from the children. In this sense, the components nuts and seeds were not included in the score and the component oil and fats only included the cooking oils and fats.

The maximum score for each component was based on its estimated relative importance with regard to risk of type 2 diabetes. A maximum score of 10 was given to breakfast, vegetables, fruit and berries, sugary drinks, whole-grain cereals and red meat components, depending on the responses from the questionnaire for dietary habits. A maximum score of 8 was given to the frequency of family meals (including breakfast, lunch and dinner eaten in the company of a friend, colleague or family member). The rest of the components, sweet snacks, salty snacks and low-fat dairy, got a maximum score of 6 except for cooking oils and fats that had a maximum score of 4. A higher score indicated higher or more frequent consumption, except for sugary drinks, red meat, sweet snacks and salty snacks where higher scores indicated lower consumption. Total HDS was calculated as the sum of the component scores. Score ranged from 0 to 86; a higher score indicated better quality of the diet and a maximum score indicated full achievement of the Feel4Diabetes intervention dietary goals.

### Biochemical analysis

Blood samples were drawn in parents after at least 8 h of overnight fasting. Each biomarker was measured in each country using the same method. The levels of total cholesterol (TC), triglycerides (TG) and HDL cholesterol (HDL-c) were determined by standard enzymatic methods. LDL cholesterol (LDL-c) levels were calculated with Friedewald’s formula when serum TG was < 400 mg/dL. Blood glucose concentration was measured with the glucose-oxidase method. Insulin levels were measured via radioimmunoassay and homeostasis model assessment of insulin resistance (HOMA–IR) was estimated as fasting serum glucose (mg/dL) × plasma insulin (μU/mL)/405. IR was considered when HOMA-IR was equal or higher than 2.5 [10].

### Statistical analysis

Normality was checked and Student’s *t* test and Mann–Whitney were applied for parametric and non-parametric distributions, respectively. Variables were transformed when needed. Descriptive study characteristics are shown as mean and standard deviations or median (min–max) if not normally distributed. Two groups were created for parents, those insulin-resistant, when HOMA-IR was higher than 2.5, and those parents who are non-insulin resistant, with equal or lower values than 2.5.

Linear regression was used to assess the association between continuous parental HOMA (independent) and children’s behaviours: physical activity (PA), screen time and HDS (dependent variables), independently. Two models were created to assess the differences depending on the variables used for adjustment and to assess the additional effect of the variables included in the model 2: a crude model including sex and age of the included parent and the child and an adjusted model that included the crude model and the child’s BMI and the parental education for adjustment.

In addition, *K*-means cluster analysis, considering HDS, PA and screen-time, was performed to identify clusters of children with similar lifestyle patterns. The *K*-means algorithm was applied with a pre-defined maximum of 100 iterations to generate separate cluster solutions for two to six clusters. In order to find a stable clustering pattern, several solutions were obtained with different starting seeds. Iterations were generated until no change in cluster centroids was observed. The stability of the final solution was examined by randomly splitting the database into half and repeating the same clustering procedure, until satisfactory results were observed (a maximum of 84 allocated to different clusters, representing 3.9% of the total sample). The *Z*-scores for each marker of lifestyle behaviour were calculated to standardize values and to avoid large differences between markers of lifestyle behaviour. The criteria to choose the clusters were based on stability of the cluster solution and interpretability.

Finally, multilevel ordinal logistic regression analyses, considering country as level due to the country-specific differences, were conducted to investigate the association between parental IR status (independent), considering non-insulin resistant as reference, and the obtained clusters of the child’s lifestyle behaviours ordered by healthiness (dependent variables). Cluster 1 was the healthiest one. Two models were created; a crude model including sex of the included parent and the child and an adjusted model that included the crude model and the child’s BMI and the parental education for adjustment.

## Results

Table 1 online shows the main characteristics of the adult participants of the study by category of IR. A higher percentage of females were allocated in the non-insulin resistance category in comparison with males (81.4% versus 18.6%). Regarding the cardio-metabolic variables, those in the insulin-resistant group had higher mean values (*p* < 0.001) for the anthropometric indices, BMI, WC, weight and height, for the blood pressure markers (SBP and DBP) and for all the biomarkers: TC, LDL-c, HDL-c, TG, glucose, insulin and HOMA (*p* < 0.001) except for HDL-c were those non-insulin resistant that had lower mean value (*p* < 0.001).

**Table 1:** Mean differences of the children’ body composition and lifestyle behaviours by parental insulin resistance^a^

	Non-insulin resistant* n = 1445		Insulin-resistant* n = 672		
	**Mean or Median**	**SD or min–max**	**Mean or median**	**SD or min–max**	***P* value**
*Age*	8.16	0.97	8.25	0.99	0.054
*Weight (kg)*	28.2	16.9–71.2	29.3	17.3–74.3	** < 0.001**
*Height (cm)*	130.3	7.82	130.9	8.06	0.125
*BMI*	16.5	11.2–32.3	17.9	11.7–31	** < 0.001**
***Lifestyle behaviours***					
***HDS total (0****–****86)***	49.03	9.44	48.62	8.94	0.338
*Breakfast component*	10	0–10	10	0–10	**0.008**
*Vegetables component*	0	0–10	0	0–10	**0.05**
*Fruits and berries*	6	0–10	6	0–10	0.238
*Sugary drinks*	8	0–10	8	1–10	0.19
*Whole-grain cereals*	1	0–10	1	0–10	0.315
*Low-fat dairy*	0	0–6	0	0–6	0.835
*Oils and fats for cooking*	4	2–4	4	2–4	0.885
*Red meat*	8	0–10	8	0–10	0.354
*Sweet snacks*	3	0–6	3	0–6	0.57
*Salty snacks*	5	0–6	5	0–6	0.429
*Family meals*	2	0–6	2	0–6	0.808
***PA (days 60 min PA)***	4.56	2.37	4.41	2.32	0.189
***Screen time (hours per week)***	13.5	0–63	15.5	0–58	**0.014**

Boldface indicates significant *p*-value (*p* < 0.05)

*BMI* body mass index, *HDS* Healthy Diet Score, *PA* physical activity

*Non-insulin resistant: HOMA less than 2.5; *Insulin-resistant*: HOMA higher or equal to 2.5

^a^Values are expressed as mean (standard deviation) or median [min–max] if not normally distributed

The mean differences of the children’s lifestyle behaviours by parental IR are shown in Table 1. Children of insulin-resistant parents had higher weight and higher BMI (*p* < 0.001) than those of non-insulin-resistant parents. There were no mean differences between children of IR parents and non-IR parents in total HDS score but those from insulin-resistant parents had a lower mean value in the breakfast component and in the vegetable component of the HDS (*p* = 0.008 and *p* = 0.05, respectively). Also, those from insulin-resistant parents spent more hours in screen activities per week than those from non-insulin-resistant parents (15.09 h versus 16.18, respectively; *p* = 0.014).

Table 2 shows the results from the linear regression between the continuous parental HOMA (independent) and the three lifestyle behaviours measured: HDS, physical activity and screen-time (dependent variables). Parental HOMA was inversely associated with HDS, but only in the crude model (*β* =  − 0.068, 95%CI =  − 0.336, − 0.073). On the other hand, parental HOMA was directly associated with screen-time in the crude model and even when adjusting by age and sex from both parent and child, with child’s BMI and parental education (*β* = 0.067, 95%CI = 0.073, 0.370).

**Table 2:** Linear regression analysis between continuous parental HOMA (independent) and each child’s lifestyle behaviour indicator^a^ (dependent)

		**Crude model**^**b**^			**Model 1**^**c**^		
**Parental HOMA**	**HDS**	***β***	**95%CI**	***p***	***β***	**95% CI**	***p***
		− 0.068	− **0.336, − 0.073**	**0.002**	− 0.039	− 0.259, 0.022	0.099
	**PA**	***β***	**95%CI**	***p***	***β***	**95%CI**	***p***
		0.005	− 0.031, 0.039	0.810	− 0.039	− 0.040, 0.039	0.101
	**Screen time**	***β***	**95%CI**	***p***	***β***	**95%CI**	***p***
		0.09	**0.142, 0.407**	** < 0.001**	0.067	**0.073, 0.370**	**0.003**

Boldface indicates significant *p*-value (*p* < 0.05)

*HDS* Healthy diet score, *HOMA* Homeostasis Model Assessment, *PA* physical activity

^a^Values are *β* values and 95% confidence intervals (CI) with *p*-values

^b^Crude model: adjusted by age and sex of the children and their parents

^c^Model 1: adjusted by age and sex of the children and their parents, body mass index of the child and parental education

**Fig. 1:**
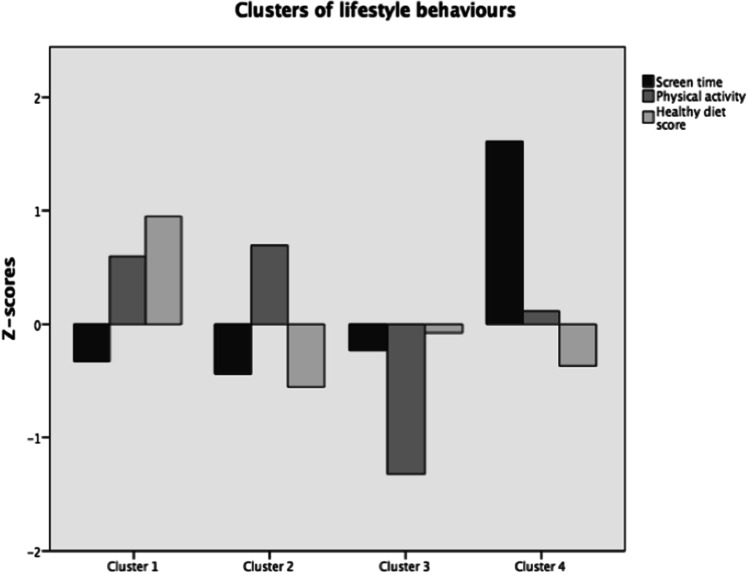
Clusters of the children’s lifestyle behaviours: screen time, physical activity and healthy diet score, according to the *z*-scores of the *k*-means obtained for each solution. Cluster 1: “healthy”, cluster 2: “active but poor HDS”, cluster 3: “low physically active” and cluster 4: “screen-timers”

Out of the *k*-means cluster analysis, several solutions were found ranging from two to four clusters. The criteria to choose the clusters were based on interpretability and the similar sample’s number found by cluster. Therefore, the solution with four clusters was chosen (Fig. 1). The clusters were labelled based on the corresponding *z*-score values for the lifestyle behaviours (Table 2 online). Cluster 1 was considered the “healthy” cluster, cluster 2 was labelled as “active but poor HDS”, cluster 3 was considered as “low physically active” and cluster 4 was considered as “screen-timers”.

Finally, Table 3 shows the association between having an insulin-resistant parent (independent) and the ordered clusters (dependent). Cluster 1 was considered as the healthiest option. The odds of being in a higher cluster (i.e. a less-healthy one) when having an insulin-resistant parent was 1.23 (95%CI: 1.034–1.479; *p* = 0.020) in the crude model. In addition, in the full adjusted model, considering also parental education and child’ BMI along with age and sex of the measured parent and the child, the odds of being in a higher cluster increased in 19% when having an insulin-resistant parent (OR: 1.19; 95%CI: 1.001–1.437; *p* = 0.049).

**Table 3:** Multilevel logistic regression between the status of insulin resistance (independent) of the parents (non-insulin resistant versus insulin-resistant) and the clusters of the children’s lifestyles (dependent)^a^

	**Cluster lifestyle**					
	**Crude model**^**b**^			**Model 1**^**c**^		
**HOMA categories**	**OR**	**95% CI**	***p***	OR	**95% CI**	***p***
Non-insulin resistant (Reference)^*^	-	-	-	-	-	-
Insulin resistant	1.23	1.034–1.479	**0.020**	1.19	1.001–1.437	**0.049**

Boldface indicates significant *p*-value (*p* < 0.05)

^a^Values are odds ratio and 95% confidence interval with *p*-values

^b^Crude model: adjusted by age and sex of the children and their parents

^c^Model 1: adjusted by age and sex of the children and their parents, body mass index of the child and parental education

*Non-insulin resistant: HOMA less than 2.5; Insulin-resistant: HOMA higher or equal to 2.5

## Discussion

In the present study, we found significant differences in the lifestyle behaviours: diet, physical activity and sedentary behaviour, and the patterns of these lifestyle behaviours of the children, depending on the presence or not of parental IR. Children of parents with IR had higher BMI and ate breakfast and vegetables less frequently compared to children of non-insulin-resistant parents. Also, higher parental HOMA was associated with more child’s screen time. Finally, the odds of being in a less-healthy cluster when having a parent with IR was 19% higher compared with those children of non-insulin-resistant parents.

Nowadays, the prevalence of IR varies across countries, ranging from 15.5 to 46.5% in adults [23, 24]. In the present study, 31.74% of the families had at least one parent with IR according to Matthews’ cut-off [10]. However, it should be noted that those included in the present study were selected based on specific criteria, a minimum score risk for developing T2D according to the FINDRISC. Thus, the prevalence in a general European population sample should be lower.

Previous studies have suggested IR differences by sex with women showing lower prevalence of IR [25]. In our sample, women had lower prevalence than men, 44.5% and 55.5%, respectively. These findings may be explained by oestrogen’s role in preventing beta-cell apoptosis, reducing pro-inflammatory signalling and improving insulin action [26]. This could, at least partly, explain the differences found in our sample.

Also, we found significant differences in body composition according to adults’ IR category as parents with IR had higher BMI than the non-insulin-resistant ones. Adipose tissue is characterized by decreased clearance of chylomicrons and impaired insulin-mediated inhibition of lipolysis, which could lead to IR [27]. Thus, there is a direct link between body composition and IR. In addition, in the present study, we have found significant mean differences in the cardio-metabolic biomarkers of the parents by the IR categories. IR is now considered as a marker of metabolic disturbances and even the primary pathophysiological event that drives other cardio-metabolic factors to cluster [28].

It is well known the association between family history of T2D and the risk of children’s diabetes [29]. Specifically, maternal T2D seems to be a potential risk factor for developing T2D in children [29]. Consequently, parents with impaired glucose or insulin metabolism, i.e. IR, could have children at risk of IR already. A previous study suggested that children of parents with IR had higher IR and obesity degree [7]. In our study, children of parents with IR also had higher BMI in comparison with those from parents with no IR. Previously, it has been observed that parental BMI correlates with their children’s [30] and this could be explained by the genetic and behavioural factors that they share in the household, among other factors. This could be explained by the genetic and behavioural factors that they share in the household. Specifically, the behavioural pattern from the parents could shape the children’s lifestyle behaviours, as it could be conditioned by the socioeconomic status, parental education, food availability, parental preferences, parental physical activity and sociocultural influences, among others [31]. It has been shown that parental obesity increases the odds of failure to therapeutic lifestyle change intervention in children and adolescents [32]. Additionally, it has been suggested that parental history of obesity could be used as a practical approach to identify children with cardio-metabolic risk [33]. Furthermore, studies have demonstrated that parental unhealthy lifestyles have a great influence on children’s obesity and lifestyles [34, 35]. Thus, previous literature suggests that there is a clear association between parental obesity and unhealthy lifestyles in child’s obesity and cardio-metabolic risk. However, there are no similar studies assessing the association between parental IR and child’s anthropometric indices, cardio-metabolic risk or lifestyle behaviours.

In the present study, children of parents with IR ate breakfast less frequently than those from no-IR parents. A meta-analysis observed that skipping breakfast is associated with a significantly increased risk of type 2 diabetes in adults [36]. Thus, those children of IR parents could probably be influenced by their parental breakfast behaviour. On the other hand, a previous study suggested that children with daily breakfast consumption had lower levels of insulin resistance [37]. So, enhancing breakfast consumption could have benefits for children and adults regarding IR. Also, children of insulin-resistant parents had a higher screen time and this behaviour in children was also associated with parental HOMA-IR. In adults, IR has been associated with physical inactivity [38], so it can be expected that their children present also higher levels of physical inactivity and sedentary behaviours. Intensive lifestyle behavioural interventions for adults with impaired glucose tolerance have shown that progression to type 2 diabetes can be reduced by half [39]. Thus, a causal link between lifestyle behaviours and diabetes risk has been suggested [40]. Also, it has been observed that meeting the recommendations for the individual lifestyle components like diet [41, 42], PA [43] or sedentary behaviours [44] is inversely associated with the risk of diabetes in adults. In the present study, those children of parents with IR had higher probabilities of an unhealthy lifestyle pattern. As parents with diabetes risk already show unhealthy lifestyles, results suggest that there is an unhealthy family environment that could lead to an increased risk even for the children, especially considering that IR and obesity may be the earliest manifestations in children of IR parents [7].

The present study has some limitations. Firstly, parents were selected from high-risk families, with a high FINDRISC score, so there was a bias as the parents were selected for being at risk and, therefore, in the prevalence of IR. Moreover, most of the participating parents were women and, as mentioned before, oestrogen could have a role improving insulin action, suggesting that in a sex-balanced sample IR prevalence could be higher. Also, data from physical activity was obtained by questionnaires and the HDS has not been validated for children. On the other hand, there are some strengths in the present study. Firstly, we used data from a large European cohort of schoolchildren of six countries and their families. Secondly, all questionnaires were developed in English then culturally adapted to each language and back translated to English and again to the local language as a quality assessment method and all the measurements were standardized across countries and performed by trained researchers. Finally, this is the first study to assess the association between parental IR and child’s lifestyle behaviours.

## Conclusion

In conclusion, children of parents with IR had higher BMI and higher screen-time than children of non-insulin-resistant parents. Screen-time was also associated with parental HOMA. This study suggests that having an insulin-resistant parent increases the probabilities of having an unhealthy lifestyle pattern in children. Thus, in those families with an insulin-resistant parent, the children’s lifestyle behaviours should be assessed as these could be associated with an unhealthy family environment. Further research is needed, using these results, to develop interventions to reduce the risk of diabetes in children in at-risk clusters.

## Supplementary information

Below is the link to the electronic supplementary material.

Supplementary file1 (DOCX 25 KB)

## Data Availability

Data not available due to ethical restrictions.

## Abbreviations

BMI: Body mass index
EBRB: Energy balance-related behaviours
HDS: Healthy Diet Score
HDL-c: High-density lipoprotein cholesterol
HOMA-IR: Homeostasis model assessment for insulin resistance
IR: Insulin resistance
LDL-c: Low-density lipoprotein cholesterol
OR: Odds ratio
PA: Physical activity
TC: Total cholesterol
TG: Triglycerides
WC: Waist circumference
