# Supplementary material for: Parental insulin resistance is associated with unhealthy lifestyle behaviours independently of body mass index in children: The Feel4Diabetes study
Source: Eur J Pediatr. 2022 Mar 26;181(6):2513–22. doi: 10.1007/s00431-022-04449-0 (PMC9110461; doi:10.1007/s00431-022-04449-0)
Supplement: Supplementary file 1 — Supplementary file1 (DOCX 25 KB) [file 431_2022_4449_MOESM1_ESM.docx]

**Supplementary table 1**. Mean differences of the cardio metabolic parameters of the parents by parental insulin resistance ^1^

|  | **Non-insulin resistant *** | | **Insulin-resistant*** | |  |
| --- | --- | --- | --- | --- | --- |
|  | **n=1445** | | **n=672** | | **p** |
| Male | 269 | 18.6% | 299 | 44.5% |  |
| Female | 1176 | 81.4% | 373 | 55.5% | **<0.001** |
| Age | 40.36 | 5.22 | 40.60 | 5.48 | 0.347 |
| BMI (kg/m2) | 26.96 | 4.87 | 32.16 | 5.51 | **<0.001** |
| WC (cm) | 89.9 | 12.62 | 104.23 | 14.1 | **<0.001** |
| Weight (kg) | 75.16 | 15.78 | 92.61 | 18.03 | **<0.001** |
| Height (cm) | 166.67 | 8.62 | 169.56 | 9.46 | **<0.001** |
| SBP (mm Hg) | 115.67 | 15.85 | 125.46 | 17.87 | **<0.001** |
| DBP (mm Hg) | 77.12 | 10.76 | 83.62 | 12.02 | **<0.001** |
| Cholesterol (mg/dL) | 191.08 | 37.41 | 201 | 9.1 | **<0.001** |
| LDL-c (mg/dL) | 116.01 | 32.39 | 124.96 | 33.2 | **<0.001** |
| HDL-c (mg/dL) | 57.24 | 14.21 | 46.75 | 12.13 | **<0.001** |
| TG (mg/dL) | 89.52 | 50.21 | 155.38 | 118.33 | **<0.001** |
| Glucose (mg/dL) | 90.3 | 10.49 | 105.3 | 30.63 | **<0.001** |
| Insulin (µIU/mL) | 41.81 | 16.96 | 122.72 | 94.36 | **<0.001** |
| HOMA | 1.34 | 0.55 | 4.66 | 4.39 | **<0.001** |

Boldface indicates significant p-value (p< 0.05).

^1^*Values are n and percentage (%) or means ± SDs or medians.*

**No insulin-resistant: HOMA less than 2.5; Insulin-resistant: HOMA higher or equal to 2.5.*

*BMI. Body mass index; DBP. Diastolic blood pressure; HDL-c. High density lipoprotein cholesterol; HOMA. Homeostatic model assessment; SBP. Systolic blood pressure; TC. Total cholesterol; TG. Triglycerides; WC. Waist circumference*

**Supplementary Table 2**. Z scores of relative consumption frequencies in the three clusters at baseline Z- scores of k-mean ^1^

|  | Cluster 1 | | Cluster 2 | | Cluster 3 | | Cluster 4 | |
| --- | --- | --- | --- | --- | --- | --- | --- | --- |
| Screen time | -0.33 | 0.02 | -0.44* | 0.02 | -0.22 | 0.02 | 1.61*†* | 0.04 |
| Physical activity | **0.61** | **0.02** | **0.69***†* | **0.017** | **-1.29*** | **0.01** | **0.11** | **0.04** |
| HDS | **0.97***†* | **0.02** | **-0.55*** | **0.027** | **-0.07** | **0.03** | **-0.36** | **0.04** |

Boldface indicates significant p-value (p< 0.005).

^1^*Values are means ± SDs (standard deviations).*

**The lowest mean value within a row*

*† The highest mean value within a row*

*HDS: healthy diet score.*
